# Comparison of the complete plastomes and the phylogenetic analysis of *Paulownia* species

**DOI:** 10.1038/s41598-020-59204-y

**Published:** 2020-02-10

**Authors:** Pingping Li, Gongli Lou, Xiaoran Cai, Bin Zhang, Yueqin Cheng, Hongwei Wang

**Affiliations:** 1 College of Life Science, Henan Agricultural University, Zhengzhou, 450002 China; 2 College of plant protection, Henan Agricultural University, Zhengzhou, 450002 China

**Keywords:** Ecology, Evolution, Molecular biology, Plant sciences

## Abstract

*Paulownia* species are important ecological, economic and ornamental species, but their phylogenetic relationship remains unclear, which seriously affects the development and utilization of these important resources. The complete chloroplast genomes of six *Paulownia* species were assembled by next-generation sequencing data. By adding two known *Paulownia* chloroplast genomes to these six assembled genomes, we performed the comparative analysis and phylogenetic tree reconstruction of *Paulownia*. The results indicated that the chloroplast genomes of *Paulownia* species ranged in size from 154,107 to 154,694 bp. These chloroplast genomes contained 117 unique functional genes, including 80 protein-coding genes, four rRNA genes, and 33 tRNA genes. Twelve hotspot regions, five protein-coding genes and seven noncoding regions, were identified in the chloroplast genomes that showed high levels of sequence variation. Additionally, positive selection was observed in three genes, *rps2*, *rbcL* and *ndhG*. The maximum likelihood (ML) and Bayesian (BI) analysis strongly supported the monophyletic origin of *Paulownia* species, which clustered into two major clades: One clade included *P. coreana*, *P. tomentosa* and *P. kawakamii*, while the other clade comprised the 5 other species including *P. fargesii* and *P. australis*. This study provides useful genetic information for phylogenetic reconstruction, taxonomic discrepancies, and studying species evolution and phylogeography in *Paulownia*.

## Introduction

Paulownia is a general term for plants from the genus *Paulownia*, which includes a total of eight species, *P. coreana*, *P. tomentosa*, *P. kawakamii*, *P. fargesii*, *P. australis*, *P. fortunei*, *P. elongata* and *P. catalpifolia*^1,2^. Paulownia originated in China and has a long history of cultivation in China. To date, it has been introduced in Japan, Australia, Brazil, Europe and the United States^3,4^. Paulownia is a fast-growing tree, and its wood has a series of excellent characteristics, such as its light weight and lack of splitting and deformation and its moisture-proof, sound-insulating, fire-resistance, and corrosion-resistance properties, which enable its use in building materials, furniture, agricultural tools, handicrafts, cultural articles and musical instruments^5–7^. In addition, Paulownia flowers, leaves, fruits, and bark can also be used as medicine, with anti-inflammatory, cough-relieving, diuretic, and antihypertensive effects^8^. Moreover, Paulownia is also an ornamental plant with lush inflorescence and various flower colors and is often used as a street tree^9^. In short, Paulownia is an important ecological, economic and ornamental tree with a wide range of uses.

Researchers have studied the genetic relationship among *Paulownia* species based on morphological characteristics and variations in DNA information, but their inferences on the evolutionary relationship of *Paulownia* species were affected by unstable morphological characteristics or insufficient genetic information, leading to significant differences between research results^10–12^. The unclear phylogenetic relationship of *Paulownia* species, especially the uncertainty of the parental source of the hybrid species, has seriously affected the further development of these important resources and hindered the progress of Paulownia breeding. Therefore, it is necessary to clarify the evolutionary relationship of *Paulownia* species in the current forest practice of Paulownia.

In most angiosperms, chloroplast DNAs are maternally inherited and do not recombine, making them suitable for the analysis of phylogenetic relationships among species, especially related species^13–15^. The evolution rate or genetic diversity of different regions of the chloroplast genome varies greatly, and the successful development of common primers in those high-variability regions make these loci widely used in the study of phylogenetic relationships among species; among these loci, *matK* and *rbcL* are most commonly used^16,17^. With the application of high-throughput sequencing technology, the cost of sequencing has been greatly reduced, which makes it possible to reveal the phylogenetic relationships among species by using the genetic information of the whole chloroplast genome in many plant groups^15,18–20^. The abundant genetic variation information and maternal genetic characteristics of the chloroplast genome are particularly suitable for reconstructing the phylogeny of low-level taxonomic hierarchies with complex relationships. Based on 1564 single-nucleotide variants in the chloroplast genome, Carbbonell-Caballero *et al*. constructed highly credible phylogenetic trees for wild and cultivated citrus, and Wambugu *et al*. also used chloroplast genome data to construct a clear pedigree relationship among wild rice species and cultivars^21,22^.

In the long process of coevolution, most of the genes of the chloroplast genome have been transferred to the nuclear genome^23^, but approximately 120 genes remain in the chloroplast genome and participate in the physiological processes of chloroplast photosynthesis, transcription and translation, making the chloroplast a semiautonomous organelle^15^. In the long process of evolution, some chloroplast genes underwent adaptive selection to the environment^24,25^. This study applied high-throughput sequencing technology to assemble chloroplast genomes of six *Paulownia* species to explore the following topics: The genetic diversity of the chloroplast genomes of *Paulownia*; the hypervariable regions of the chloroplast genomes of *Paulownia*; the loci in the chloroplast genome involved in adaptive selection during the evolution of *Paulownia*; and the phylogenetic relationships of the eight *Paulownia* species.

## Results and Analysis

### Molecular features of the chloroplast genomes

**Figure 1:**
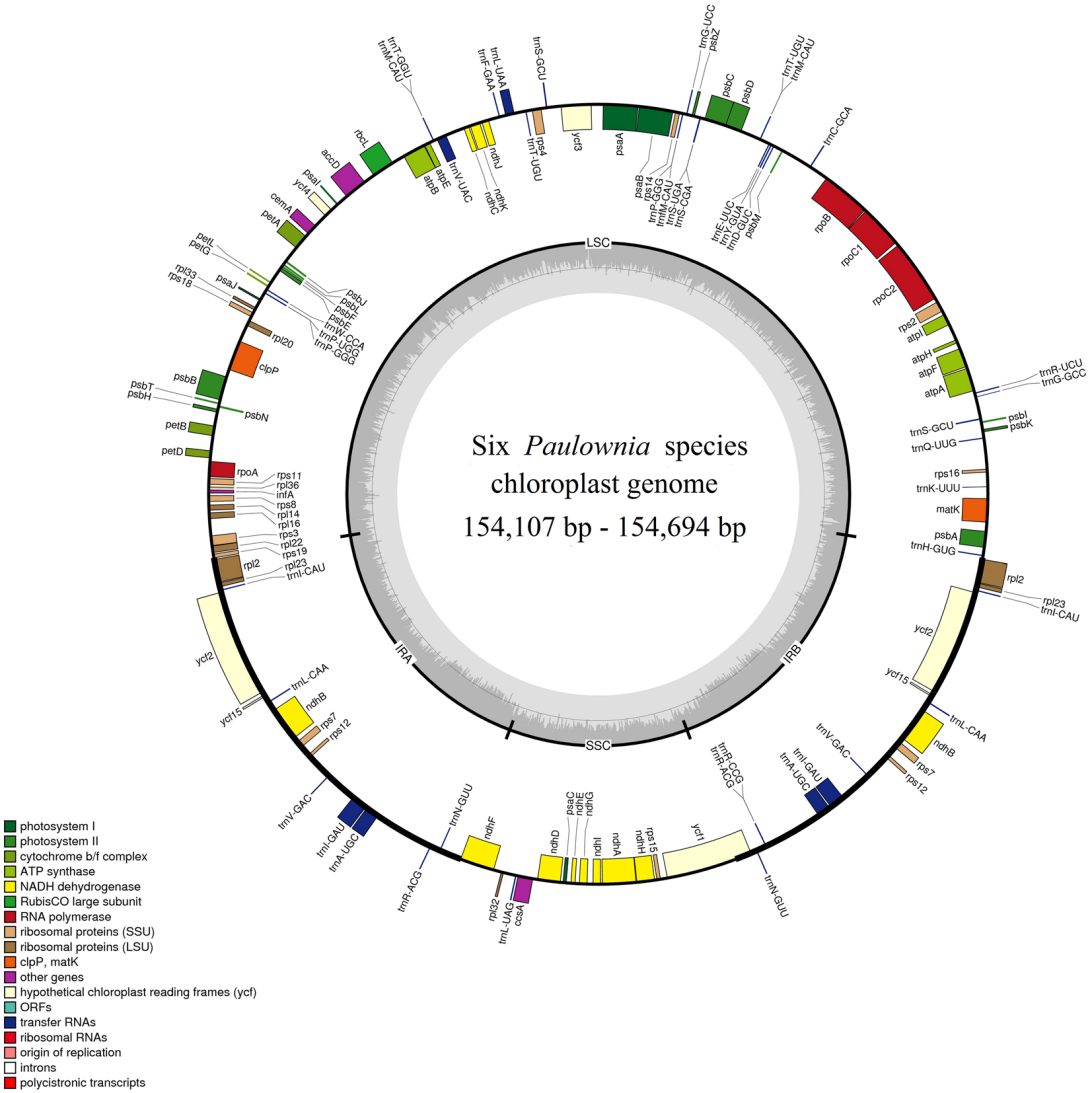
Map of the chloroplast genome of the six *Paulownia* species. Genes drawn inside the circle are transcribed clockwise, and those outside the circle are transcribed counterclockwise. The dashed gray area in the inner circle shows the percent GC content of the corresponding genes. LSC, SSC, and IR denote large single copy, small single copy, and inverted repeats, respectively. The circular map of the chloroplast genome was drawn using OGDRAW 1.3.1 (http://ogdraw.mpimp-golm.mpg.de/).

The chloroplast genome lengths of six *Paulownia* species ranged in size from 154,107 bp for *P. kawakamii* to 154,694 bp for *P. catalpifolia* (Fig. 1 and Table 1). As in most land plants, the six *Paulownia* plastid genomes exhibited a typical quadripartite structure consisting of a large single-copy region (LSC; 84,807–85,420 bp) and a small single-copy region (SSC; 17,731–17,740 bp) separated by two inverted repeats (IRs; 51,540–51,560 bp). These genomes had similar GC contents, with values from 37.96% to 37.99%, consistent with other previously reported *Paulownia* chloroplast genomes^26^. In addition, the gene content, order, and orientation were identical in the chloroplast genomes of the six *Paulownia* species. They contained 117 unique functional genes, including 80 protein-coding genes, four rRNA genes, and 33 tRNA genes (Supplementary Table 1). Among these genes, 17 genes were duplicated, with six protein-coding genes, four rRNAs, and seven tRNAs. In addition, seventeen of the genes contained one or two introns (Supplementary Table 1).

**Table 1:** Comparison of the chloroplast genome features of the eight *Paulownia* species.

Species	*P. elongata*	*P. australis*	*P. kawakamii*	*P. fargesii*	*P. catalpifolia*	*P. fortunei*	*P. coreana*	*P. tomentosa*
Accession number	MK618176	MK618177	MK618178	MK618179	MK618180	MK618181	KP718622	KP718624
Total chloroplast genome size (bp)	154,688	154,247	154,107	154,692	154,694	154,676	154,545	154,540
LSC (bp)	85,415	84,972	84,807	85,418	85,420	85,400	85,241	85,236
IR (bp)	51,540	51,544	51,560	51,540	51,540	51,540	51,568	51,568
SSC (bp)	17,733	17,731	17,740	17,734	17,734	17,736	17,736	17,736
Total number of genes	134	134	134	134	134	134	134	134
Protein-coding genes	86	86	86	86	86	86	86	86
rRNAs	8	8	8	8	8	8	8	8
tRNAs	40	40	40	40	40	40	40	40
GC content (%)	37.97%	37.99%	37.99%	37.96%	37.97%	37.97%	38.00%	38.00%

### Sequence variation

A total of 216 single-nucleotide polymorphisms (SNPs) were detected among the chloroplast genomes of the eight *Paulownia* species, of which 97 were base transitions, accounting for 44.9% of the total base mutations, and the remaining 119 mutations were base transversions, accounting for 55.1% of the total mutations (Table 2). Of these mutations, T-A was the most common mutation with 61 occurrences, followed by C-T and G-A with 49 and 48 occurrences, respectively; C-G was the least common with only five occurrences.

**Table 2:** Nucleotide mutation type.

	Nucleotide mutation	Number of mutations
Conversion	A-G	48
	T-C	49
Transversion	A-C	29
	A-T	61
	T-G	24
	C-G	5
Total		216

**Figure 2:**
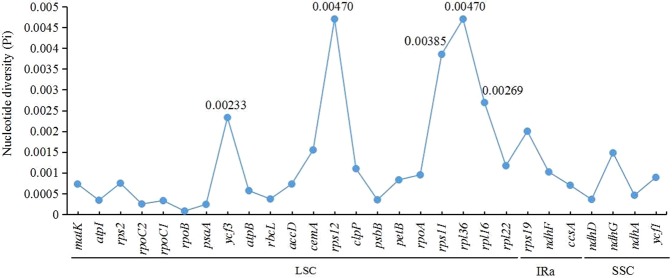
The nucleotide diversity of coding regions among the eight *Paulownia* species.

**Figure 3:**
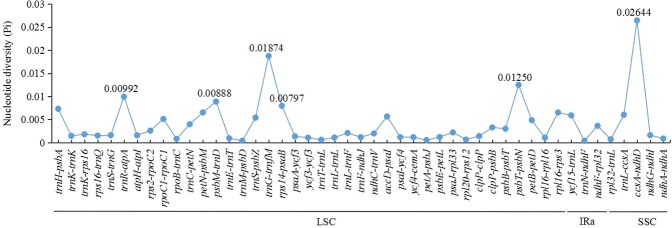
The nucleotide diversity of nocoding regions among the eight *Paulownia* species.

Sequence divergence analysis indicated that there was a low level of nucleotide diversity (Pi = 0.00066) across the eight *Paulownia* species. IR regions were the most conserved feature, with the lowest Pi value of 0.00012; the SSC region had relatively high sequence variation, with a Pi value of 0.00106; and the LSC region showed a medium Pi value of 0.00089 (Table 3). In addition, the Pi value (0.00032) in the coding regions was lower than that (Pi = 0.00102) in the noncoding regions (Table 3). In the coding regions, the greatest variability was detected in the genes *rps12* and *rpl36* with Pi values of 0.0047; other genes with Pi values above 0.00200 were *rps11*, *rpl16* and *ycf3* (Fig. 2). In the noncoding regions, seven regions (*ccsA-ndhD*, *trnG-trnfM*, *psbT-psbN*, *trnR-atpA*, *psbM-trnD*, *rps14-psaB*, and *trnH-psbA*) showed high levels of sequence variation (Pi ≥ 0.00731); among them, the region *ccsA-ndhD* had the highest Pi (0.02644) (Fig. 3). These hotspot regions could be used as potential markers for species identification and molecular breeding within this genus in the future.

**Table 3:** The values of nucleotide diversity (Pi) in different regions among the eight *Paulownia* species.

	Structural region			Noncoding region	Coding region
	LSC	IRa	SSC		
Total number of sites	83,984	25,763	17,716	75,487	79,429
Number of polymorphic sites	165	6	38	161	55
Pi values	0.00089	0.00012	0.00106	0.00102	0.00032
Theta-W	0.00076	0.00009	0.00083	0.00084	0.00027

### Repeat sequence variation

**Figure 4:**
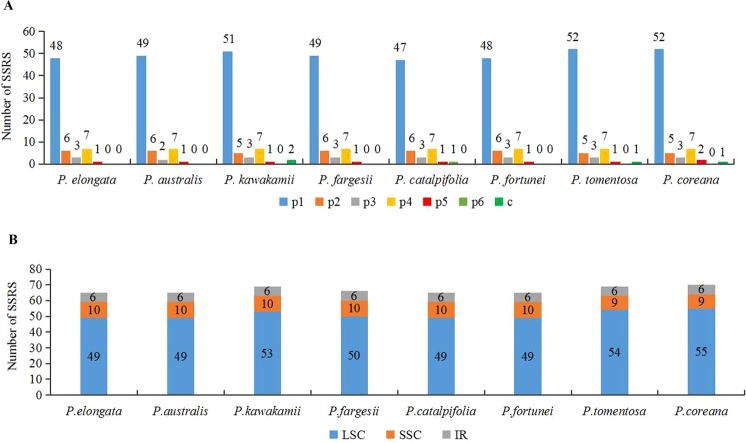
Analyses of repeated sequences in the chloroplast genomes of the eight *Paulownia* species. (**A**) The number of repeats in the eight *Paulownia* chloroplast genomes. p1: Mono. p2: Di. p3: Tri. p4: Tetra. p5: Penta. p6: Hexa. c: Imperfect repeat. (**B**) Distribution of SSRs in the eight *Paulownia* chloroplast genomes.

Among the chloroplast genomes of the eight *Paulownia* species, seven simple sequence repeats (SSR) types were identified, mononucleotide, dinucleotide, trinucleotide, tetranucleotide, pentanucleotide, hexanucleotide and compound (Fig. 4a). The eight *Paulownia* species contained similar numbers of SSRs. The maximum number was 70 in *P. coreana*, and the minimum was 65 in *P. elongata*, *P. australis*, *P. catalpifolia* and *P. fortunei*. The mononucleotide repeat was the most common type of microsatellite, and tetranucleotide motifs were the second most abundant in the *Paulownia* plastomes. For example, in *P. tomentosa*, there were 69 SSRs, of which 52 were mononucleotide repeats with a ratio of 75%, seven were tetranucleotide repeats, five were dinucleotide repeats, three were trinucleotide repeats. A/T repeats were the most common mononucleotides with ratios ranging from 93.9% to 96.2% in the eight *Paulownia* species, while AT/TA repeats were the most abundant dinucleotide with ratios of 100%; other SSR types had only one to two copies (Supplementary Table 2). Most SSR loci were located in the LSC region. Among the eight *Paulownia* species, 49 to 55 SSR loci were in the LSC region, and only six and ten (or nine) SSRs were distributed in the IR and SSC regions, respectively (Fig. 4b).

### Positive selection analysis

To determine which genes in the chloroplast genome of *Paulownia* were involved in adaptive evolution, we conducted a neutral test of protein-coding genes with genetic variation by calculating the ratio (dN/dS) of the nonsynonymous to synonymous substitution. The results indicated that 3 protein-coding genes were subject to positive selection (dN/dS > 1). These genes under positive selection exhibited functional diversity, including one NADPH dehydrogenase subunit gene (*ndhG*), one ribosomal protein gene (*rps2*) and one RuBisCO gene (*rbcL*).

### Phylogenetic relationships of the eight *Paulownia* species

**Figure 5:**
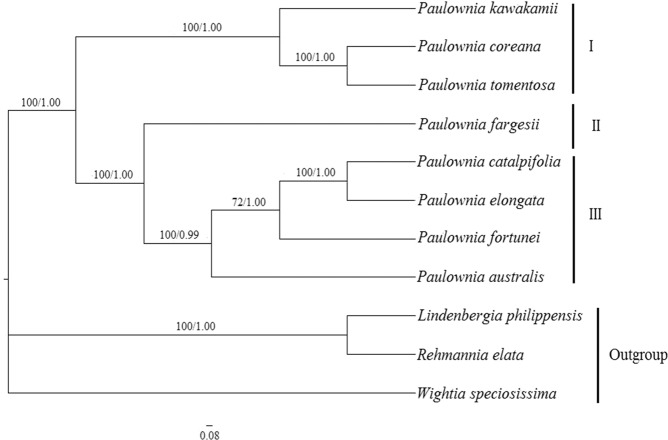
Phylogenetic tree based on whole plastomes with three positively selected genes being removed. The numbers to the left of the slashes on the braches show the bootstrap values obtained by maximum likelihood analyses, and those to the right show the posterior probabilities according to Bayesian inference.

The robust phylogenetic relationships of *Paulownia* species were reconstructed based on entire chloroplast genome sequences with three positively selected genes being removed using *Wightia speciosissima*, *Rehmannia elata* and *Lindenbergia philippensis* as outgroups (Fig. 5). The maximum likelihood (ML) and Bayesian (BI) analysis strongly suggested that the eight *Paulownia* species formed a monophyletic group, and these species clustered into two major clades, a small clade and a large clade, with 100% bootstrap values and 1.00 posterior probabilities, respectively. The small clade (I) included *P. coreana*, *P. tomentosa* and *P. kawakamii*, in which the closely related *P. coreana* and *P. tomentosa* form a sister branch with *P. kawakamii*. In another clade, *P. fargesii* was the earliest species to diverge, forming one subclade (II). The remaining four species clustered into the second subclade (III) with high bootstrap support, in which *P. australis* was sister to the combined clade of the other three species. In the combined clade, *P. elongata* was most closely related to *P. catalpifolia*.

## Discussion

### Genetic diversity of the chloroplast genome in different plant groups

Because the divergent time and the rate of chloroplast genome evolution are different in different plant groups, the genetic diversity of chloroplast genome in these plant groups is quite different. According to morphological and ecological data combined with geological records, the following results were obtained: In the early tertiary period, there was only one species of *Paulownia*, and it was divided into two primitive species of *Paulownia*, *P. kawakamii* and *P. tomentosa*, in the Miocene period. Later, other species of *Paulownia* were formed through evolution and hybridization^27^. Our results showed that the nucleotide polymorphism (Pi value) of the *Paulownia* chloroplast genomes was only 0.00066, which is significantly lower than that of many other groups. The Pi value of the chloroplast genome of 5 *Rosa* species was 0.00154, with a nucleotide polymorphism 3 times that of the genus *Paulownia*^28^. The average Pi value of the chloroplast genome of 6 species of *Ipomoea* was 0.0045, nearly 10 times the Pi value of the chloroplast genome of *Paulownia*^29^; the chloroplast genome of *Aristolochia* has a higher nucleotide polymorphism than the chloroplast genome of *Paulownia*, and its Pi was 0.01717, which is 31 times that of *Paulownia*^20^.

### Genetic diversity in different chloroplast regions

The genetic polymorphisms in different regions of the chloroplast genome vary substantially. In general, the single copy (SC) regions (containing LSC and SSC regions) of the chloroplast genome have higher genetic diversity than the IR regions in most plant groups^30^. In our study, the Pi values of SSC and LSC in the *Paulownia* chloroplast genomes were 0.00104 and 0.00089, respectively, both of which were significantly higher than the Pi value (0.00012) in the IR regions. Similar results were also found in other plant groups. The Pi values of LSC and SSC in the chloroplast genomes of *Aristolochia* were 0.02182 and 0.03114, respectively, which were also much higher than the Pi value (0.00411) in the IR regions^20^. The difference in genetic diversity among regions of the chloroplast genome also appeared at the family level. The IR regions of Apiaceae species were far more conserved than the SC regions, with an average Pi value of 0.002 for the former and 0.009 for the latter^31^. The percentage of nucleotide variation in the SC sequences (12.7%) was also higher than that in IRs (4.14%) in the chloroplast genomes of 6 Adoxaceae species^32^. However, the opposite was found in some groups. For example, in Caprifoliaceae chloroplast genomes, the percentage of nucleotide variation in the SC regions (17.61%) was slightly lower than that in the IR regions (21.25%)^32^.

The coding region of the chloroplast genome is more conserved due to its functional limitations; therefore, the genetic diversity of the coding region is lower than that of the noncoding region. The genetic diversity (Pi = 0.00102) of the noncoding region was significantly higher than that of the coding region (Pi = 0.00033) in *Paulownia* chloroplast genomes, which was consistent with the results of other groups with ratio differences. The genetic polymorphism of the noncoding region was 3.1 times that of the coding region in *Paulownia*, 3.9 times that in six Adoxaceae species, 3.5 times that in eight Caprifoliaceae species and 2.4 times that in six *Ipomoea* species^29,32^. Because of their abundant nucleotide variations, which can provide rich genetic information, noncoding regions are often employed to analyze the phylogenetic relationship of species and probe into plant evolution and colonization^33–35^. Many studies have shown that genetic diversities also differ greatly among noncoding regions of the chloroplast genome, and the regions with the greatest variation are usually called hotspot regions^20^. In different plant groups, hotspot regions vary. Dong *et al*. compared the chloroplast genomes of 29 plant species from 12 genera and identified 19 noncoding regions with high variability, of which *pl32-rnL* and *trnH-psbA* had the highest genetic variation^14^. The most variable noncoding regions included *trnH-GUG-psbA, trnR-UCU-atpA, trnC-GCA-petN, ycf3-trnS-GGA*, and *trnL-UAA-trnF-GAA* in six Adoxaceae chloroplast genomes^32^; and *TrnN-GUU-ndhF* is the hotspot region in *Capsicum*^36^. The regions with the highest percentage of sequence variation were *ccs-trnL-UAG, psbI-trnS-GCU, rpl32-ndhF, trnT-UGU-TrnL-UAA* and *petN-psbM* in *Echinacea*^19^. In three closely related East Asian wild roses, *matK-trnK, psbI-trnS-trnG, rps16-trnG, rpoB-trnC* and *rps4-trnT* were the most divergent intergenic regions, with Pi values exceeding 0.006^28^.

There were also significant differences in the degree of variation among chloroplast protein-coding regions. Some coding regions show high variability in most plant groups, such as *ycf1, nahF, rbcL*, and *matK*, which are often used for barcoding^14^. Other coding regions show high polymorphism only in some groups, such as *trnK*, *rpl22*, *ndhI*, *clpP*, and *rps16*^14,32^. In the chloroplast genomes of *Paulownia*, the high-polymorphism coding regions included *rpl36*, *rps12*, *rps11*, *rpl16*, and *ycf3*, most of which are genes that encode ribosomal proteins.

In short, although many universal primers for chloroplast DNA have been used, the overall variation in the chloroplast genome of target groups should be detected before selecting certain DNA fragments for further research because of the difference in hotspot regions in different plant groups. The hypervariable loci found in *Paulownia* in this study, including coding regions and noncoding regions, can provide abundant variation information, which can be used to identify *Paulownia* species and study species differentiation, population genetics and phylogeography.

### Gene selective analysis

Chloroplasts are organelles that carry out photosynthesis in green plants and are the most abundant energy converters on earth. Some enzymes and structural proteins within chloroplasts are encoded by genes of chloroplast genomes^24^. During chloroplast genome evolution, most genes were subjected to purifying selection due to functional limitations; some of these genes were involved in adaptation to the environment and underwent positive selection, while others were under neutral evolution. By calculating the ratio of dN to dS (dN/dN) for the coding genes with genetic variation, we identified 3 genes (*rps2*, *rbcL* and *ndhG*) under positive selection in the chloroplast genomes of *Paulownia*, and each of three selected genes performed different physiological functions. A few genes undergoing positive selection also occurred in some other plant groups. Five plastid genes (*rbcL, clpP, atpF, ycf1* and *ycf2*) were subject to positive selection in 7 *Panax* species^30^, and only three chloroplast genes (*clpP, ycf1* and *ycf2*) underwent positive selection in the chloroplast genomes of seven Sileneae species^37^. In many other groups, multiple chloroplast genes show a positive selection effect. One-third of the chloroplast genes in PACMAD grasses, 27 genes in the genus *Iodes*, 19 genes in Dipsacales species, and 10 genes in *Gossypium* evolved under positive selection^25,32,38,39^. Those identified selected genes may be underwent certain functional diversification during their evolutionary history.

Among the selected chloroplast genes in *Paulownia*, the *rbcL* gene encodes the large subunit of RuBisCO, which plays an important role in plant photosynthesis. Previous studies showed that *rbcL* is often under positive selection because of being the target of selection in relation to the changes in temperature, drought and carbon dioxide concentration^24,30,32,39^. So, the *rbcL* gene could be a positively selected site during the evolutionary process of *Paulownia*. The *ndhG* gene is another selected gene in *Paulownia*. In higher plants, chloroplast NAD(P)H dehydrogenase can protect plants from photoinhibition or photooxidation stress caused by strong light and alleviate the decrease in the photosynthetic rate and growth delay caused by drought^40,41^. This enzyme has important functions and is composed of many subunits. Due to adaptations to the environment, some of the genes encoding these subunits (*ndh*) are involved in adaptive evolution and exhibit positive selection^25,38,42^. For example, in Australian *Citrus*, *ndhF* exhibited a positive selection effect for its involvement in the adaptation to hot and dry climates^21,43^, and *ndhG* were also subjected to positive selection in *Iodes*^38^. The positive selection signal of *ndhG* in the *Paulownia* genus might be the result of adaptation to different environments because the climate of the growth areas of different *Paulownia* species is different.

### Phylogenetic relationships of *Paulownia* species

Due to the frequent hybridization among *Paulownia* species, there is a general genetic introgression among these species, which leads to a complex phylogenetic relationship of *Paulownia* species^11^. Although the phylogenetic relationships of *Paulownia* species have been investigated based on morphological, structural, physiological, biochemical and genetic information, a reliable phylogenetic tree for *Paulownia* species has not been established. Using the complete chloroplast genome information, we constructed a highly reliable pedigree tree of *Paulownia*. In our study, the *Paulownia* genus was of monophyletic origin, and its eight species clustered into two clades. *P. coreana*, *P. tomentosa* and *P. kawakamii* formed one clade, while the five other species of the genus formed another clade. Our results were generally consistent with those obtained based on the morphological traits of *Paulownia*. Fan selected 22 independent traits to conduct comparative analysis of *Paulownia* species^9^. According to the Q cluster of these morphological traits, he concluded that *P. elongata*, *P. catalpifolia* and *P. fortunei* were clustered together, forming a white flower Paulownia group with other species, while *P. tomentosa* and *P. kawakamii* were included in another Paulownia group. In addition, some of our results are also supported by studies based on molecular data. For example, by analyzing random amplified polymorphic DNA (RAPD) data, Lu *et al*. categorized *P. fargesii*, *P. australis*, *P. catalpifolia* and *P. fortunei* into one group^44^.

The systematic positions of *P. fargesii* and *P. australis* have always been the most controversial issue. Fan’s study indicated that *P. fargesii*, *P. tomentosa* and *P. kawakamii* clustered into one clade, while *P. australis* formed a separate clade^9^. The phylogenetic relationship established by Mo based on inter simple sequence repeat (ISSR) data suggested that *P. fargesii* and *P. australis* were also divided into two different groups^10^. However, according to morphological traits, Xiong *et al*. proposed that *P. fargesii* and *P. australis* were closely related to *P. tomentosa* and *P. kawakamii*, and all four species were classified into one group^45^. In our study, *P. fargesii* and *P. australis* form a large clade together with *P. elongata*, *P. catalpifolia* and *P. fortunei* with high bootstrap support.

Based on the above analysis, it is very clear that, in the *Paulownia* genus, *P. coreana*, *P. tomentosa* and *P. kawakamii* form an evolutionary branch, while *P. fortunei*, *P. elongata* and *P. catalpifolia* are involved in forming another branch. In addition, in our study, the most controversial systematic positions of *P. fargesii* and *P. australis* have been well resolved.

## Materials and Methods

### Extraction, genome sequencing and assembly of the plant materials

Fresh leaves of six *Paulownia* species, *P. kawakamii*, *P. fargesii*, *P. australis*, *P. fortunei*, *P. elongata* and *P. catalpifolia*, were collected from different provinces in China (Supplementary Table 3). The genomic DNA, which was extracted by using the modified CTAB method, was used to construct a library with an inserted fragment ~270 bp in size and was sequenced according to the strategy of 150 bp paired-end reads on the Illumina HiSeq. 2500 platform. The sequencing depth was 20 × . After that, six genomic libraries were established. Clean data, which were obtained after filtering raw data, were assembled by SOAPdenovo_v2.04 (http://soap.genomics.org.cn/soapdenovo.html)^46^ according to the chloroplast genome sequence of *P. tomentosa* (KP718624). The optimal assembly results were obtained after adjusting the multiple parameters repeatedly, and then GapCloser_v1.12: (http://soap.genomics.org.cn/soapdenovo.html) was used to fill gaps. The boundaries of LSC-IR and SSC-IR were validated using PCR-based sequencing. Primers were designed by Primer Premier 5.0. The complete chloroplast genome sequences of the six species were deposited in GenBank, accession numbers are MK618176- MK618181 (Table 1).

### Genome annotation

The protein-coding sequences and noncoding RNAs of the complete chloroplast genome were predicted using the software DOGMA (http://dogma.ccbb.utexas.edu/)^47^ and revised based on the referential chloroplast genome of *P. tomentosa* and the start and stop codons.

The coding genes were homologously aligned using BLAST^48^ in different databases, including NR (http://www.ncbi.nlm.nih.gov/), KEGG (http://www.genome.jp/kegg/), COG (http://www.ncbi.nlm.nih.gov/COG/), GO (http://geneontology.org/) and Swiss-Prot (http://www.ebi.ac.uk/uniprot/) databases for functional annotation. Finally, the circular maps of the six chloroplast genomes were drawn using OGDRAW 1.3.1(http://ogdraw.mpimp-golm.mpg.de/)^49^.

### Comparative analysis

We compared the whole chloroplast genome sequences of *Paulownia* species using Geneious, and then DnaSP version 5.1^50^ was used to calculate the Pi value, the SNP sites in the eight *Paulownia* chloroplast genomes and the nucleotide substitutions in the coding regions of the eight *Paulownia* genomes. The values of dN and dS for each protein-coding exon with genetic variation were calculated using the codeml package (seqtype = 1, model = 0) in PAMLX^51^. The SSRs in the eight *Paulownia* chloroplast genomes were identified using MISA with the parameters set to ten repeat units for mononucleotide SSRs, five repeat units for dinucleotide, four repeat units for trinucleotide, and three repeat units for tetranucleotide, pentanucleotide, and hexanucleotide SSRs. The imperfect repeat sequences were limited to interruptions between 2 SSRs that did not exceed 10 bp.

### Phylogenetic analysis

The complete chloroplast genomes of the eight *Paulownia* species, *W. speciosissima*, *R. elata* (NC_034312) and *L. philippensis* (NC_022859) were aligned by MAFFT in Geneious V.9.1^52^. The complete chloroplast genome sequences of *W. speciosissima*, *R. elata* and *L. philippensis* were included as the outgroups downloaded from NCBI. Phylogenetic trees were constructed by maximum like lihood (ML) and Bayesian analysis (BI) methods using chloroplast genome sequences with positively selected genes being removed. ML analyses were performed using RAxML-HPC BlackBox v.8.2.10 with the GTR + G model and 1,000 bootstrap replicates with the CIPRES Science Gateway website^53,54^. BI was performed with MrBayes 3.2.6^55^ with the following settings: Markov chain Monte Carlo simulations for 1,000,000 generations with four incrementally heated chains, starting from random trees and sampling one out of every 1,000 generations. The first 25% of the trees were regarded as burn-ins. The ML tree and BI tree were visualized using FigTree version 1.4.2^56^.

## Supplementary information

Supplementary information.
