# Supplementary material for: Comparison of the complete plastomes and the phylogenetic analysis of Paulownia species
Source: Sci Rep. 2020 Feb 10;10:2225. doi: 10.1038/s41598-020-59204-y (PMC7010769; doi:10.1038/s41598-020-59204-y)
Supplement: Supplementary file 1 — Supplementary information. [file 41598_2020_59204_MOESM1_ESM.pdf]

Comparison of the complete plastomes and the phylogenetic analysis of *Paulownia* species

Pingping Li, Xiaoran Cai, Bin Zhang, Gongli Lou, Yueqin Cheng & Hongwei Wang

Supplementary Table 1. Genes in the chloroplast genomes of the six *Paulownia* species

Category of genes	Group of genes	Names of genes				
Photosynthesis-related genes	Photosystem I	*psaA*	*psaB*	*psaC*	*psaI*	*psaJ*
		*ycf3***	*ycf4*			
	Photosystem II	*psbA*	*psbB*	*psbC*	*psbD*	*psbE*
		psbF	*psbH*	*psbI*	*psbJ*	*psbK*
		psbL	*psbM*	*psbN*	*psbT*	*psbZ*
	NADPH dehydrogenase	*ndhA**	*ndhB**	*ndhC*	*ndhD*	*ndhE*
		ndhF	*ndhG*	*ndhH*	*ndhI*	*ndhJ*
		ndhK				
	ATP synthase	*atpA*	*atpB*	*atpE*	*atpF**	*atpH*
		atpI				
	Rubisco	*rbcL*				
	cytochrome b/f complex	*petA*	*petB**	*petD**	*petG*	*petL*
		petN				
	cytochrome c synthesis	*ccsA*				
Transcription- and translation-related genes	ribosomal proteins	*rps2*	*rps3*	*rps4*	*rps7*	*rps8*
		rps11	*rps12***	*rps14*	*rps15*	*rps16*
		rps18	*rps19*	*rpl2**	*rpl14*	*rpl16**
		rpl20	*rpl22*	*rpl23*	*rpl32*	*rpl33*
		rpl36				
	transcription	*rpoA*	*rpoB*	*rpoC1**	*rpoC2*	
	translation initiation factor	*infA**				
RNA genes	transfer RNA	*trnA-UGC**	*trnC-GCA*	*trnD-GUC*	*trnE-UUC*	*trnF-GAA*
		trnG-GCC	*trnG-UCC*	*trnH-GUG*	*trnI-CAU*	*trnI-GAU**
		*trnK-UUU**	*trnL-UAG*	*trnL-UAA**	*trnL-CAA*	*trnfM-CAU*
		trnM-CAU	*trnN-GUU*	*trnP-UGG*	*trnP-GGG*	*trnQ-UUG*
		trnR-ACG	*trnR-UCU*	*trnS-GCU*	*trnS-UGA*	*trnS-GGA*
		trnT-UGU	*trnT-GGU*	*trnV-UAC**	*trnV-GAC*	*trnW-CCA*
		trnY-GUA				
	ribosomal RNA	*rrn5*	*rrn4.5*	*rrn16*	*rrn23*	

Supplementary Table 1. (Continued)

Other genes	RNA processing	*matK*		
	fatty acid synthesis	*accD*		
	carbon metabolism	*cemA*		
	proteolysis	*clpP***		
Genes of unknown function	conserved reading frames	*ycf1*	*ycf2*	*ycf15*

* Gene containing a single intron, ** Gene containing tow introns.

Supplementary Table 2. Type of SSRs in the chloroplast genomes of the eight *Paulownia* species

	Mono	Di	Tri	Tetra	Penta	Hexa	Imperfect repeat
P. elongata	(A)$_{10-13}$ = 24<br>(T)$_{10-12}$ = 23<br>(G)$_{11}$ = 1<br>(C)$_{11}$ = 1	(TA)$_{5-7}$ = 4<br>(AT)$_{5}$ = 2	(TTA)$_{4}$ = 1<br>(TTC)$_{4}$ = 1<br>(ATA)$_{4}$ = 1	(ATTG)$_{3}$ = 1<br>(GTCT)$_{3}$ = 1<br>(TCTA)$_{3}$ = 1<br>(GAAA)$_{3}$ = 1<br>(AAAC)$_{3}$ = 1<br>(AATA)$_{3}$ = 1	(TATTT)$_{3}$ = 1		
P. australis	(A)$_{10-13}$ = 23<br>(T)$_{10-13}$ = 23<br>(G)$_{10-11}$ = 2<br>(C)$_{11}$ = 1	(TA)$_{5-7}$ = 4<br>(AT)$_{5}$ = 2	(TTC)$_{4}$ = 1<br>(ATA)$_{4}$ = 1	(ATTG)$_{3}$ = 1<br>(GTCT)$_{3}$ = 1<br>(TCTA)$_{3}$ = 1<br>(AAAC)$_{3}$ = 1<br>(GAAA)$_{3}$ = 2<br>(AATA)$_{3}$ = 1	(TATTT)$_{3}$ = 1		
P. kawakamii	(A)$_{10-14}$ = 23<br>(T)$_{10-13}$ = 26<br>(G)$_{11}$ = 1<br>(C)$_{11}$ = 1	(TA)$_{5-6}$ = 4<br>(AT)$_{5}$ = 1	(ATA)$_{4}$ = 1<br>(TTA)$_{4}$ = 1<br>(TTC)$_{4}$ = 1	(ATTG)$_{3}$ = 1<br>(GTCT)$_{3}$ = 1<br>(AAAC)$_{3}$ = 1<br>(GAAA)$_{3}$ = 1<br>(TCTA)$_{3}$ = 1<br>(TTTC)$_{3}$ = 1<br>(AATA)$_{3}$ = 1	(TATTT)$_{3}$ = 1		(GAAA)$_{3}$tttctc(T)$_{10}$= 1<br>(T)$_{10}$c(T)$_{10}$ = 1
P. fargesii	(A)$_{10-13}$ = 23<br>(T)$_{10-12}$ = 24<br>(G)$_{11}$ = 1<br>(C)$_{11}$ = 1	(TA)$_{5-6}$ = 4<br>(AT)$_{5}$ = 2	(TTA)$_{4}$ = 1<br>(ATA)$_{4}$ = 1<br>(TTC)$_{4}$ = 1	(ATTG)$_{3}$ = 1<br>(GTCT)$_{3}$ = 1<br>(AATA)$_{3}$ = 1<br>(TCTA)$_{3}$ = 1<br>(GAAA)$_{3}$ = 2<br>(AAAC)$_{3}$ = 1	(TATTT)$_{3}$ = 1		
P. catalpifolia	(A)$_{10-13}$ = 23<br>(T)$_{10-13}$ = 22<br>(G)$_{11}$ = 1<br>(C)$_{11}$ = 1	(TA)$_{5-7}$ = 4<br>(AT)$_{5}$ = 2	(TTA)$_{4}$ = 1<br>(ATA)$_{4}$ = 1<br>(TTC)$_{4}$ = 1	(ATTG)$_{3}$ = 1<br>(GTCT)$_{3}$ = 1<br>(TCTA)$_{3}$ = 1<br>(GAAA)$_{3}$ = 2<br>(AATA)$_{3}$ = 1<br>(AAAC)$_{3}$ = 1	(TATTT)$_{3}$ = 1	(TTCAAT)$_{3}$<br>= 1	
P. fortunei	(A)$_{10-13}$ = 23<br>(T)$_{10-14}$ = 23<br>(G)$_{11}$ = 1<br>(C)$_{11}$ = 1	(TA)$_{5-7}$ = 4<br>(AT)$_{5}$ = 2	(TTA)$_{4}$ = 1<br>(TTC)$_{4}$ = 1<br>(ATA)$_{4}$ = 1	(ATTG)$_{3}$ = 1<br>(GTCT)$_{3}$ = 1<br>(AAAC)$_{3}$ = 1<br>(AATA)$_{3}$ = 1<br>(TCTA)$_{3}$ = 1<br>(GAAA)$_{3}$ = 2	(TATTT)$_{3}$ = 1		

Supplementary Table 2. (Continued)

P. tomentosa	$(A)_{10-14}$ = 24<br>$(T)_{10-13}$ = 26<br>$(G)_{10}$ = 1<br>$(C)_{10}$ = 1	$(TA)_{5-8}$ = 4<br>$(AT)_{5}$ = 1	$(TTA)_{4}$ = 1<br>$(TTC)_{4}$ = 1<br>$(ATA)_{4}$ = 1	$(ATTG)_{3}$ = 1<br>$(GTCT)_{3}$ = 1<br>$(TCTA)_{3}$ = 1<br>$(AAAC)_{3}$ = 1<br>$(GAAA)_{3}$ = 1<br>$(AATA)_{3}$ = 1<br>$(TTTC)_{3}$ = 1	$(TATTT)_{3}$ = 1	$(GAAA)_{3}$tttctc$(T)_{12}$= 1
P. coreana	$(A)_{10-14}$ = 24<br>$(T)_{10-13}$ = 26<br>$(G)_{10}$ = 1<br>$(C)_{10}$ = 1	$(TA)_{5-8}$ = 4<br>$(AT)_{5}$ = 1	$(TTA)_{4}$ = 1<br>$(TTC)_{4}$ = 1<br>$(ATA)_{4}$ = 1	$(ATTG)_{3}$ = 1<br>$(GTCT)_{3}$ = 1<br>$(TCTA)_{3}$ = 1<br>$(AAAC)_{3}$ = 1<br>$(AATA)_{3}$ = 1<br>$(GAAA)_{3}$ = 1	$(TATAT)_{3}$ = 1<br>$(TATTT)_{3}$ = 1	$(GAAA)_{3}$tttctc$(T)_{12}$ = 1

Supplementary Table 3. Sample information about the six *Paulownia* species

No.	Species	Locality	Longitude (°E)	Latitude (°N)	Altitude	Habitat
1	*Paulownia australis*	Nanping, Fujian, China	118°32´	26°41´	512.00 m	Natural forest
2	*Paulownia fargesii*	Wuhan, Hubei, China	114°18´	30°28´	46.00 m	Natural forest
3	*Paulownia fortunei*	Zhengzhou, Henan, China	113°34´	34°42´	83.00 m	Paulownia germplasm resource nursery
4	*Paulownia kawakamii*	Zhengzhou, Henan, China	113°34´	34°42´	83.00 m	Paulownia germplasm resource nursery
5	*Paulownia elongata*	Zhengzhou, Henan, China	113°34´	34°42´	83.00 m	Paulownia germplasm resource nursery
6	*Paulownia catalpifolia*	Zhengzhou, Henan, China	113°34´	34°42´	83.00 m	Paulownia germplasm resource nursery
